# Supplementary material for: Lysosomal proteomics reveals mechanisms of neuronal APOE4-associated lysosomal dysfunction
Source: Autophagy. 2025 Nov 7;21(12):3240–65. doi: 10.1080/15548627.2025.2576613 (PMC12758178; doi:10.1080/15548627.2025.2576613)
Supplement: Supplemental Figures Final R5 EK.docx [file KAUP_A_2576613_SM9711.docx]

# Supplemental Figures

| 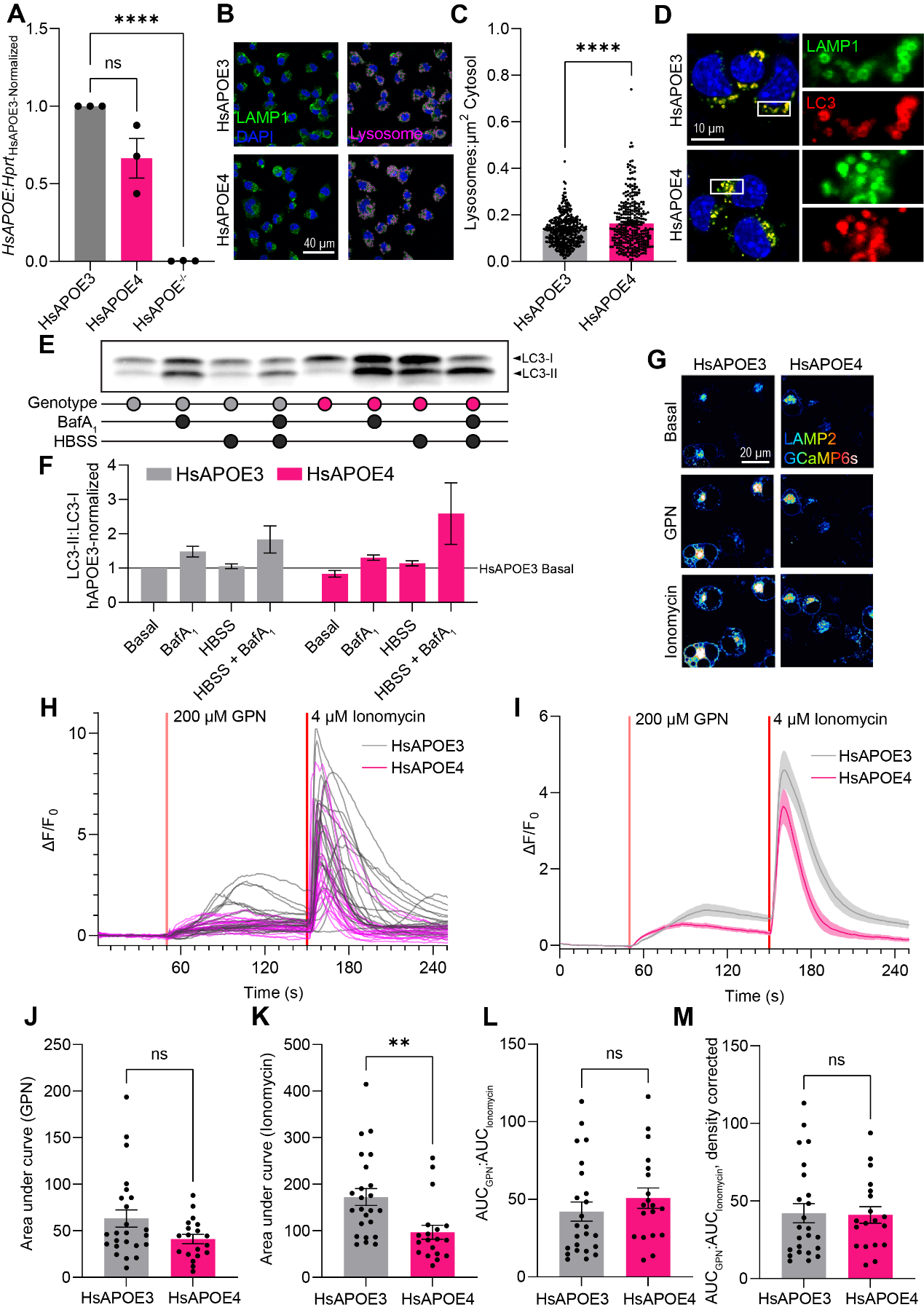 |
| --- |
| **Figure S1.** Lysosomal calcium content. (**A**) *HsAPOE* gene expression across Neuro-2a cell lines, measured by RT-qPCR. (**B**) Immunofluorescence of Neuro-2a cells expressing HsAPOE3 or HsAPOE4, stained for the integral lysosomal membrane protein LAMP1. Right panels show puncta identification using Fiji. (**C**) Lysosomal density per cell based on images such as those in panel B. (**D**) Immunofluorescence of LAMP1 and LC3 shows lysosome-autophagosome fusion in both HsAPOE3 and HsAPOE4 Neuro-2a cells following 3 h HBSS starvation and BafA_1_ treatment. (**E**) Representative western blot of LC3-I and LC3-II in HsAPOE3 and HsAPOE4 Neuro-2a cells. (**F**) Quantification of LC3-II:LC3-I ratio, normalized to internal HsAPOE3 controls, across three independent replicates. (**G**) LAMP2-GCaMP6s lysosomal calcium measurements in Neuro-2a cells shown at basal states, and upon sequential stimulation using 200 µM GPN to release lysosomal calcium, and 4 µM ionomycin to release total calcium. (**H**) Single-cell traces for lysosomal calcium release, as shown in panel D. (**I**) Mean traces of lysosomal calcium release, alongside SEM error bar traces. (**J**) Lysosomal calcium signals, as measured by the area under the curve following GPN and Ionomycin stimulation. (**K**) Total calcium sensitivity of HsAPOE3 and HsAPOE4 cells, as measured by the area under the curve following Ionomycin stimulation. (**L**) Relative lysosomal calcium release, normalized to the calcium sensitivity. (**M**) Relative lysosomal calcium release normalized to the lysosomal density, as calculated in panel C, estimates the lysosomal calcium content of each individual lysosome. |
| 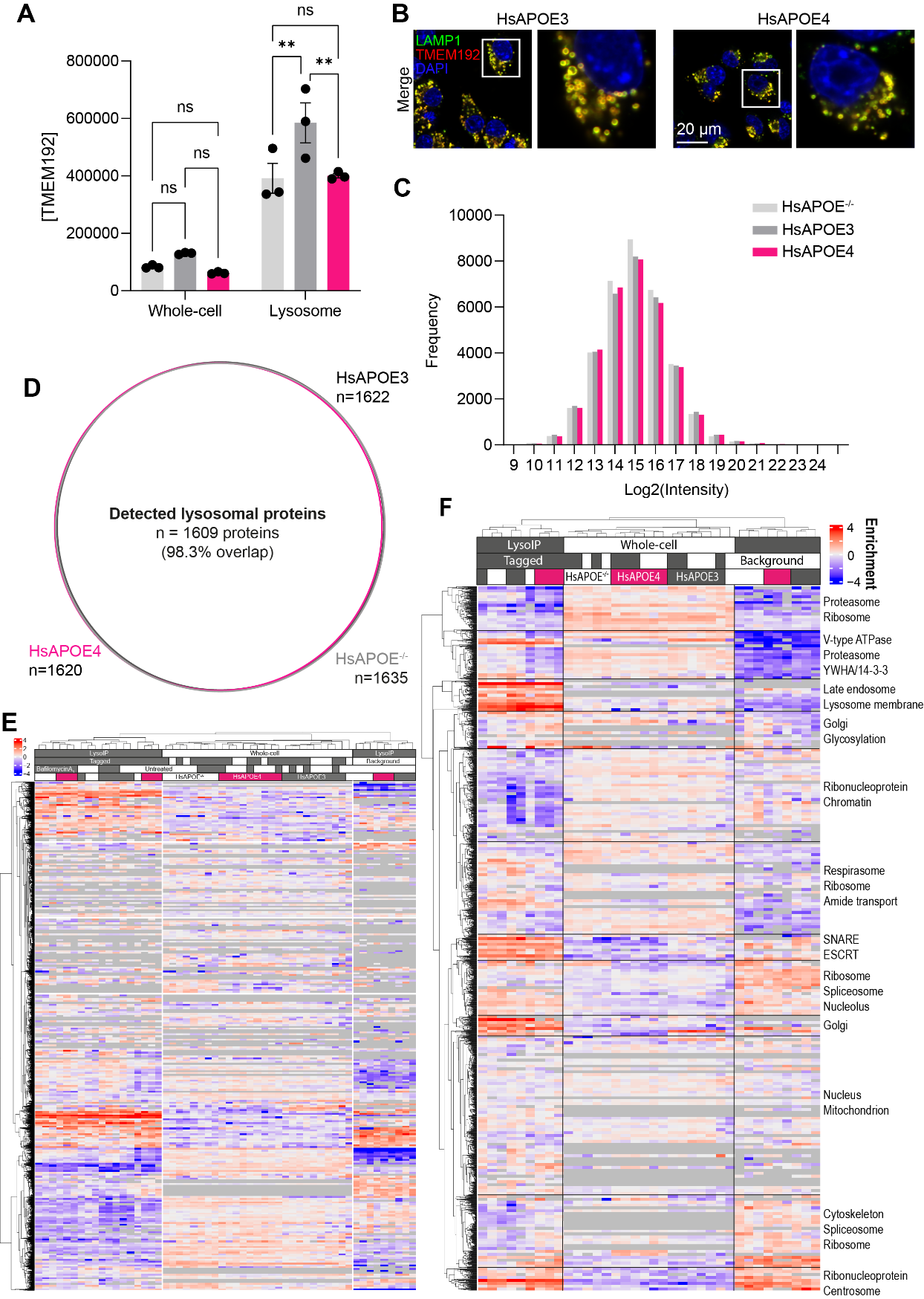 |
| **Figure S2.** Abundance proteome coverage and clustering across HsAPOE genotypes. (**A**) Human lysosomal TMEM192 tag intensities in whole-cell and lysosomal fractions of untreated Neuro-2a cells from 3 independent experiments. (**B**) Immunofluorescence of lysosomal tag and endogenous lysosomal LAMP1 across LysoIP-tagged HsAPOE3 and HsAPOE4 cells. (**C**) Histogram of Log2-transformed protein group intensities across all obtained Neuro-2a lysosomal proteomes, stratified by genotype. (**D**) Venn diagram of lysosome-enriched proteins detected in LysoIP samples across genotypes. (**E**) Full heat-map generated from raw intensities of all detected proteins across all samples reveals distinct clustering of LysoIP, whole-cell, and LysoIP background datasets, as well as sub-clustering based on Bafilomycin treatment and HsAPOE genotype. (**F**) Heat map excluding BafA_1_-treated samples, including cellular component annotations of detected proteins. Groups of data were analyzed in GraphPad Prism by 2-way ANOVA followed *post-hoc* by Dunnett’s multiple comparisons test. **P<0.01. |

| 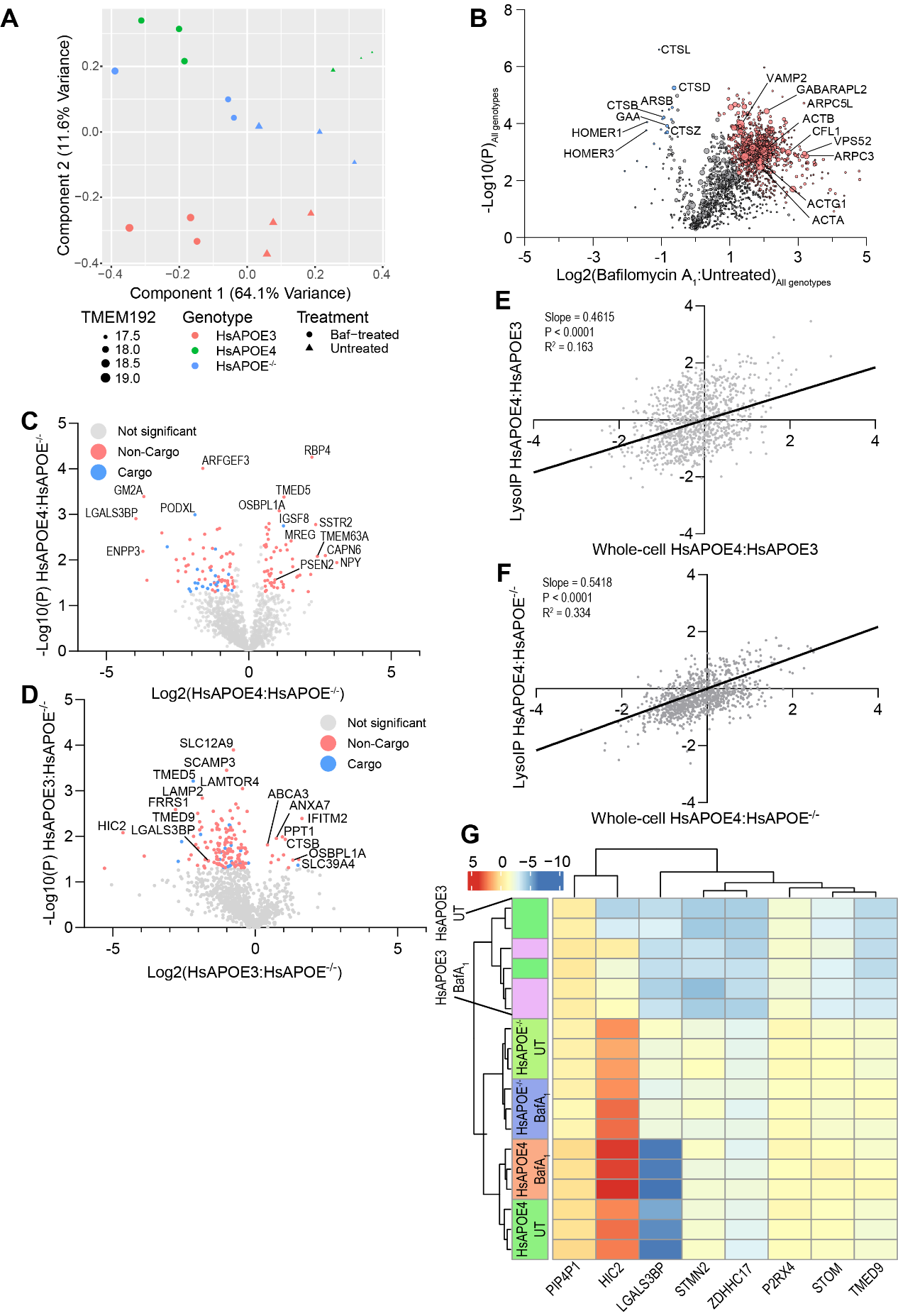 |
| --- |
| **Figure S3.** Proteomic comparisons of HsAPOE lysosomes. (**A**) Principal component analysis of all lysosomal proteomes separates samples based on Bafilomycin treatment (component 1) and HsAPOE genotype (component 2). (**B**) Volcano plot comparing BafA_1_ and untreated lysosomal proteomes, pooled across HsAPOE genotypes. Node sizes indicate relative protein abundances following BafA_1_ treatment. (**C-D**) Volcano plot comparing HsAPOE4 and HsAPOE^-/-^(C) or HsAPOE3 and HsAPOE^-/-^ (D) lysosomal proteomes. Proteins significantly changed in HsAPOE4 lysosomes are colored by their status as a lysosomal cargo protein, based on basal (red) or BafA_1_-dependent (blue) lysosomal enrichment. (**E-F**) Comparison of HsAPOE4-associated changes to whole-cell and lysosomal protein abundances to HsAPOE3 (E) and HsAPOE^-/-^ (F) proteomes, showing positive correlation between changed whole-cell and lysosomal protein levels. Only proteins detected across four sample sets (genotype and fraction) were be plotted. (**G**) Partial least squares-discriminant analysis of lysosomal proteomes reveals proteins separating experimental groups. In particular, HsAPOE4 was accompanied by lysosomal LGALS3BP depletion. |
|  |
|  |

| \| 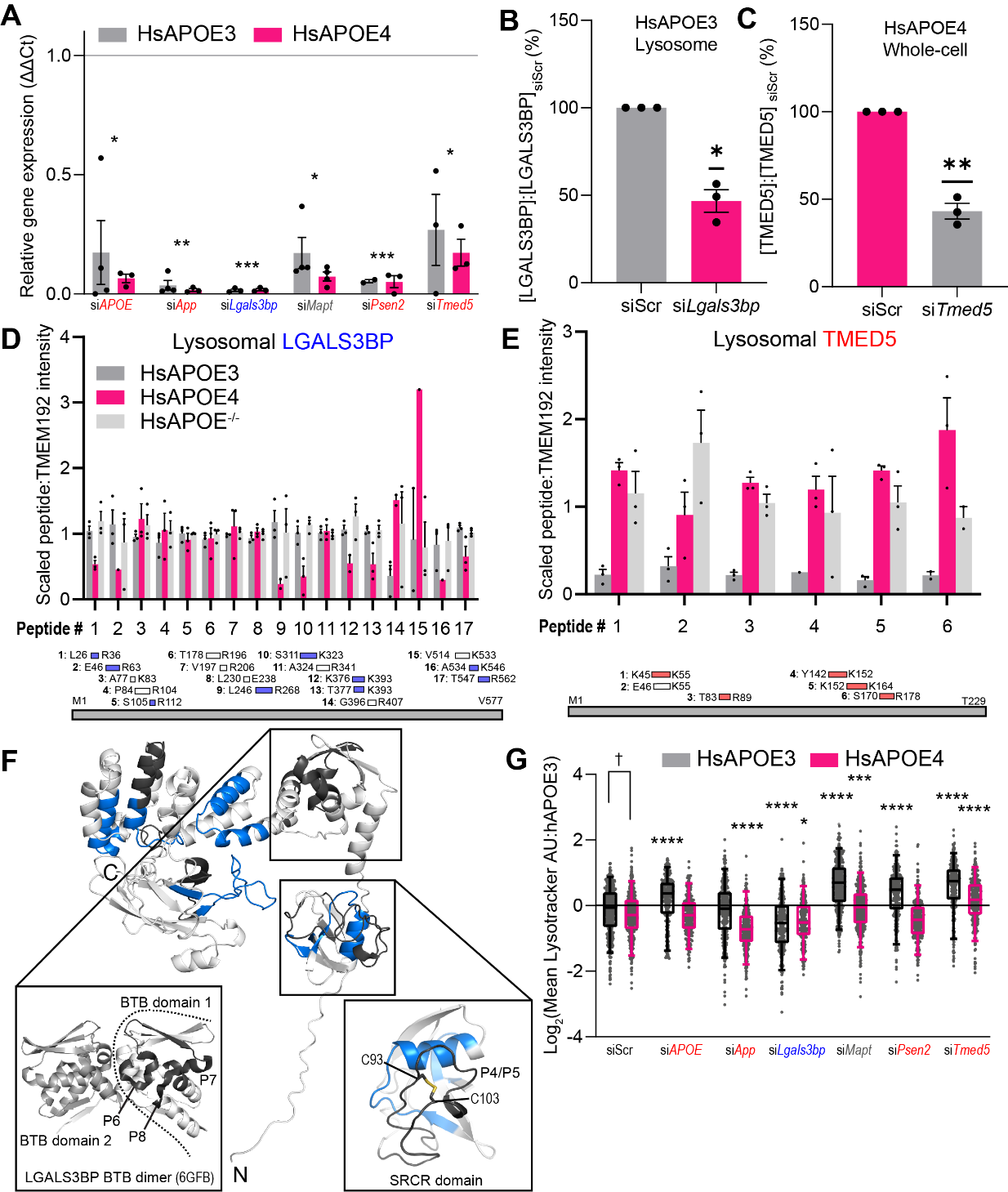 \| \| --- \| \| **Figure S4.** Identification of proteomic drivers underlying HsAPOE4 lysosomal defects. (**A**) Gene expression upon targeting indicated genes with siRNAs in Neuro-2a cells. Expression level changes are indicated relative to *Hprt1* expression and siScr-treated cells. (**B**) Efficiency of LGALS3BP protein reduction in HsAPOE3 lysosomes upon Lgals3bp siRNA knockdown relative to scrambled siRNA controls as measured by mass spectrometry. Lysosomal LGALS3BP was measured since it was undetected in the whole-cell lysate upon knockdown, precluding calculation of its knockdown efficiency. (**C**) Efficiency of TMED5 protein reduction in HsAPOE4 whole-cell lysate upon Tmed5 siRNA knockdown relative to scrambled siRNA controls as measured by mass spectrometry. (**D**) Lysosomal peptide intensities of LGALS3BP, normalized to sample TMEM192 abundance and scaled to the median intensity of each peptide across Neuro-2a HsAPOE genotypes. The cartoon below the bar chart indicates LGALS3BP peptides which are lower in HsAPOE4 lysosomes relative to both controls, denoted by blue boxes. (**E**) Lysosomal peptide intensities of TMED5, normalized to sample TMEM192 abundance and scaled to the median intensity of each peptide across Neuro-2a HsAPOE genotypes. The cartoon below the bar chart indicates TMED5 peptides which are higher in HsAPOE4 lysosomes relative to both controls, denoted by red boxes. (**F**) LGALS3BP peptides affected by HsAPOE4, mapped to AlphaFold structure AF-Q07797-F1. Peptides depleted in HsAPOE4 Neuro-2a cells are colored blue, while persistent peptides are colored grey. Peptides are labeled based on their numerical designations from panel D. Persistent peptides in the SRCR domain (P4, P5) and BTB domain dimer (P6-8, dimer structure retrieved from PDB 6GFB) are highlighted. (**G**) Influence of gene silencing on LysoTracker staining, normalized to HsAPOE3 LysoTracker intensity. Knockdown comparisons are performed relative to the same genotype siScr control. Dagger (†) comparison shows difference between HsAPOE3 and HsAPOE4 cells. Data points indicate individual cells from three independent experiments. Groups of data were analyzed in GraphPad Prism by 2-way ANOVA followed *post-hoc* by Dunnett’s multiple comparisons test. Pairs of data were analyzed by t-tests. *P<0.05, **P<0.01, ***P<0.001, ****P<0.0001. \| |
| --- | --- | --- |

| **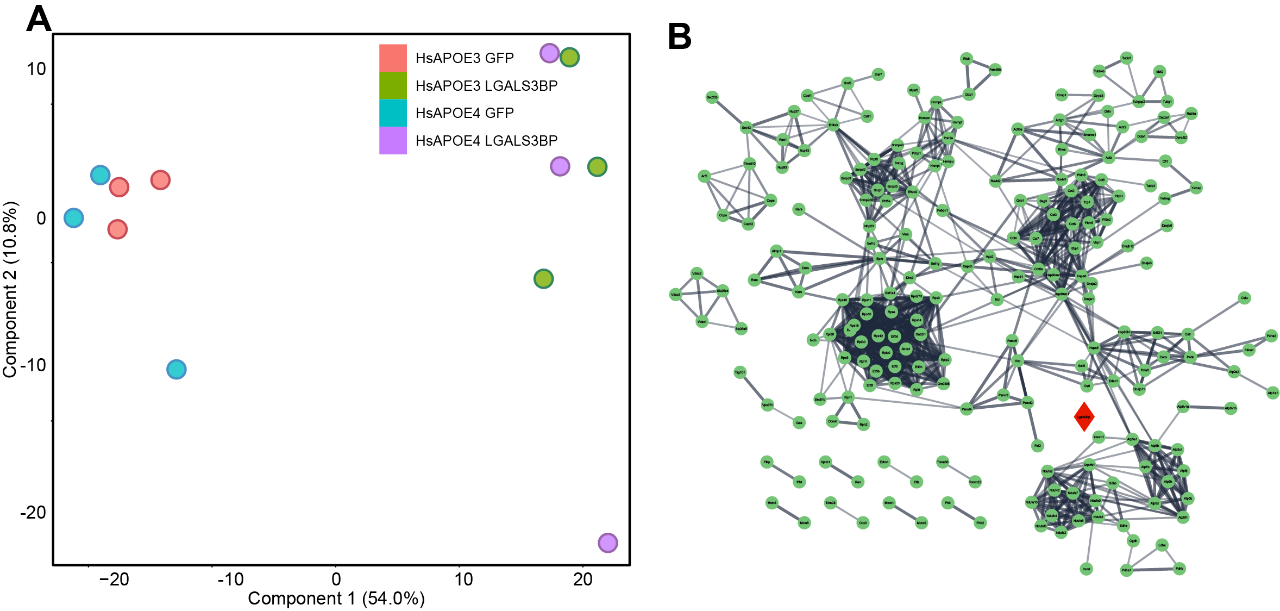** |
| --- |
| **Figure S5.** The neuronal LGALS3BP interactome. (**A**) Principal component analysis of affinity-purified proteomes shows clustering of LGALS3BP-GFP-purified samples distinct from GFP background samples (component 1), and slight separation of HsAPOE3 and HsAPOE4 samples (component 2). (**B**) Physical STRING subnetwork of identified LGALS3BP interactors. LGALS3BP is highlighted in red. |

| 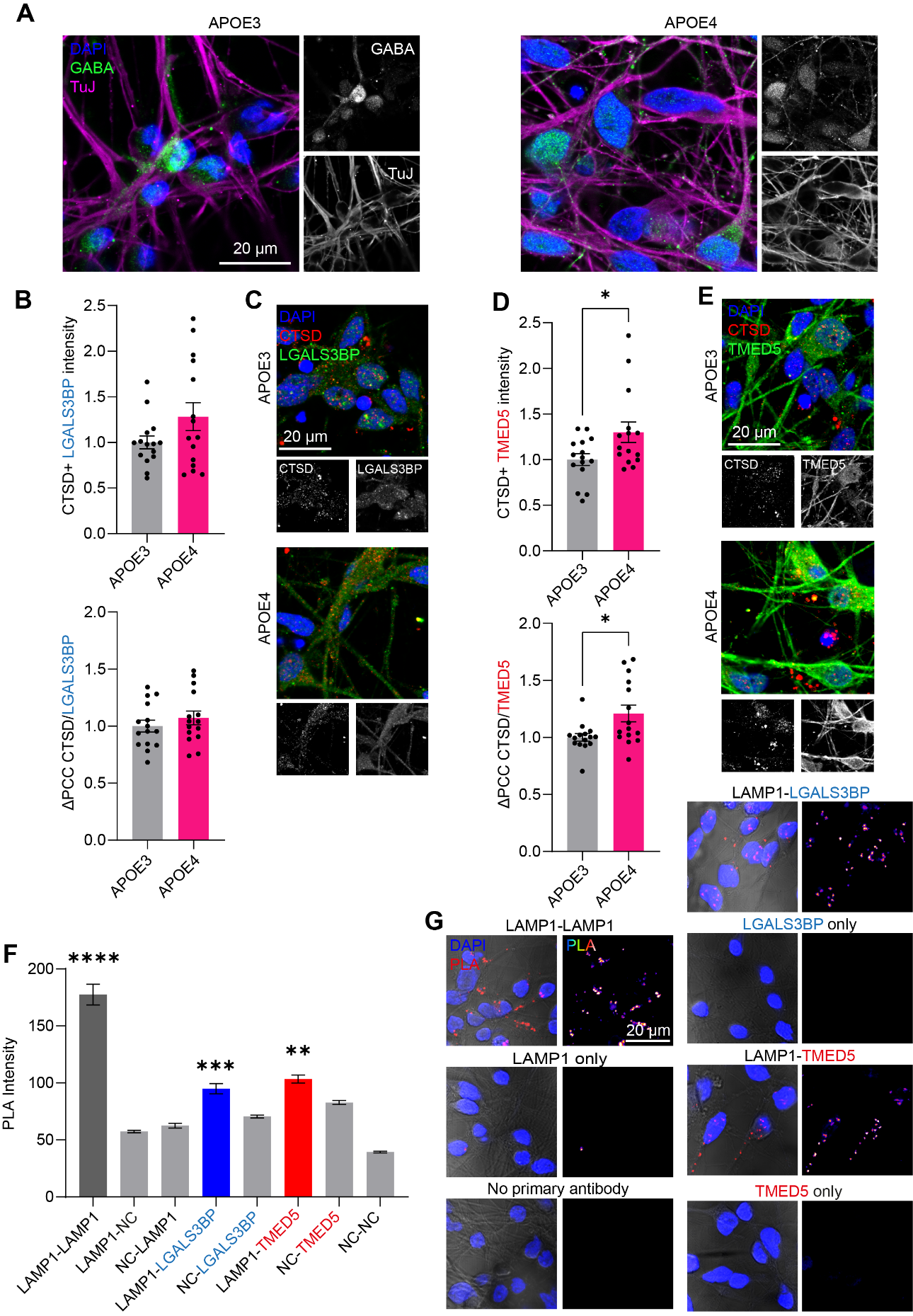 |
| --- |
| **Figure S6.** Controls for iPSC-derived neuron proximity ligation assay. (**A**) Immunofluorescence of neuronal markers following 49 days of differentiation, revealing pure cultures of TuJ^+^ neurons including GABA^+^ interneurons for both genotypes. (**B**) Quantification of difference in LGALS3BP intensity within APOE4 CTSD^+^ lysosomes (top) and change in the LGALS3BP-CTSD PCC r value (bottom), relative to APOE3 iPSC-derived neurons. (**C**) Representative images for quantification shown in panel B. (**D**) Quantification of difference in TMED5 intensity within APOE4 CTSD^+^ lysosomes (top) and change in the TMED5-CTSD PCC r value (bottom), relative to APOE3 iPSC-derived neurons. (**E**) Representative images for quantification shown in panel C. Five images from three independent experiments were analyzed by a two-tailed unpaired t-test. (**F**) Establishing PLA sensitivities, showing significant signals above single antibody negative controls (NC) for LAMP1-LAMP1, LAMP1-LGALS3BP, and LAMP1-TMED5 assays. (**G**) Representative images for quantification shown in panel F. Individual cells from three independent experiments were analyzed by a one-way ANOVA followed by a *post-hoc* Bonferroni multiple comparison test between dual antibody samples and single-antibody controls; the least statistically significant comparison is highlighted. *P<0.05, **P<0.01, ***P<0.001, ****P<0.0001. |
